# Extended MRI-based PET motion correction for cardiac PET/MRI

**DOI:** 10.1186/s40658-024-00637-z

**Published:** 2024-04-06

**Authors:** Mueez Aizaz, Jochem A. J. van der Pol, Alina Schneider, Camila Munoz, Robert J. Holtackers, Yvonne van Cauteren, Herman van Langen, Joan G. Meeder, Braim M. Rahel, Roel Wierts, René M. Botnar, Claudia Prieto, Rik P. M. Moonen, M. Eline Kooi

**Affiliations:** 1 CARIM, Cardiovascular Research Institute Maastricht, Maastricht University, Maastricht, The Netherlands; 2 Department of Radiology and Nuclear Medicine, Maastricht University Medical Center+, Maastricht, The Netherlands; 3 School of Biomedical Engineering and Imaging Sciences, King’s College London, London, UK; 4 Department of Medical Physics and Devices, VieCuri Medical Centre, Venlo, The Netherlands; 5 Department of Cardiology, VieCuri Medical Centre, Venlo, The Netherlands; 6 Escuela de Ingeniería, Pontificia Universidad Católica de Chile, Santiago, Chile; 7 Millenium Institute for Intelligent Healthcare Engineering iHEALTH, Santiago, Chile; 8 Instituto de Ingeniería Biológica y Médica, Pontificia Universidad Católica de Chile, Santiago, Chile

**Keywords:** PET/MRI, Motion correction, 2-Dimensional image navigator, Respiratory belt, Binning, Signal-to-noise ratio

## Abstract

**Purpose:**

A 2D image navigator (iNAV) based 3D whole-heart sequence has been used to perform MRI and PET non-rigid respiratory motion correction for hybrid PET/MRI. However, only the PET data acquired during the acquisition of the 3D whole-heart MRI is corrected for respiratory motion. This study introduces and evaluates an MRI-based respiratory motion correction method of the complete PET data.

**Methods:**

Twelve oncology patients scheduled for an additional cardiac ^18^F-Fluorodeoxyglucose (^18^F-FDG) PET/MRI and 15 patients with coronary artery disease (CAD) scheduled for cardiac ^18^F-Choline (^18^F-FCH) PET/MRI were included. A 2D iNAV recorded the respiratory motion of the myocardium during the 3D whole-heart coronary MR angiography (CMRA) acquisition (~ 10 min). A respiratory belt was used to record the respiratory motion throughout the entire PET/MRI examination (~ 30–90 min). The simultaneously acquired iNAV and respiratory belt signal were used to divide the acquired PET data into 4 bins. The binning was then extended for the complete respiratory belt signal. Data acquired at each bin was reconstructed and combined using iNAV-based motion fields to create a respiratory motion-corrected PET image. Motion-corrected (MC) and non-motion-corrected (NMC) datasets were compared. Gating was also performed to correct cardiac motion. The SUV_max_ and TBR_max_ values were calculated for the myocardial wall or a vulnerable coronary plaque for the ^18^F-FDG and ^18^F-FCH datasets, respectively.

**Results:**

A pair-wise comparison showed that the SUV_max_ and TBR_max_ values of the motion corrected (MC) datasets were significantly higher than those for the non-motion-corrected (NMC) datasets (8.2 ± 1.0 vs 7.5 ± 1.0, *p* < 0.01 and 1.9 ± 0.2 vs 1.2 ± 0.2, *p* < 0.01, respectively). In addition, the SUV_max_ and TBR_max_ of the motion corrected and gated (MC_G) reconstructions were also higher than that of the non-motion-corrected but gated (NMC_G) datasets, although for the TBR_max_ this difference was not statistically significant (9.6 ± 1.3 vs 9.1 ± 1.2, *p* = 0.02 and 2.6 ± 0.3 vs 2.4 ± 0.3, *p* = 0.16, respectively). The respiratory motion-correction did not lead to a change in the signal to noise ratio.

**Conclusion:**

The proposed respiratory motion correction method for hybrid PET/MRI improved the image quality of cardiovascular PET scans by increased SUV_max_ and TBR_max_ values while maintaining the signal-to-noise ratio.

*Trial registration* METC162043 registered 01/03/2017.

**Supplementary Information:**

The online version contains supplementary material available at 10.1186/s40658-024-00637-z.

## Introduction

The development of simultaneous PET/MRI has opened new avenues toward early diagnosis and improved risk stratification and treatment evaluation of patients with cardiovascular disease. The high soft tissue contrast of MRI combined with the molecular or metabolic information provided by PET makes this imaging modality well-suited for the comprehensive assessment of the myocardium. The simultaneous nature of the examination improves co-registration between PET and MR images. PET/MRI is also more convenient for the patient since both scans are performed in a single session as opposed to separate PET and MRI scans while delivering a lower dose of ionizing radiation as compared to PET/CT [1]. Lastly, since the PET and MRI examinations are performed simultaneously, it provides the opportunity to use MRI-derived motion fields to reconstruct motion-corrected PET data [2].

Cardiac imaging and coronary artery imaging is challenging due to respiratory and cardiac motion. Due to the small size and the tortuosity of the coronary arteries, preferably isotropic sequences with a high spatial resolution are needed, which leads to long scan times [3]. Therefore, coronary MR angiography (CMRA) sequences are typically acquired during free breathing. Most commonly, a one-dimensional (1D) diaphragmatic navigator [4] is used to determine the position of the lung-liver interface just before the acquisition window or, alternatively, external sensors, such as a respiratory belt, are applied to track respiratory motion [5]. Subsequently, the MR images are reconstructed based on data that are acquired in the end expiration phase. Typically, only about 30% of the data can be used for image reconstruction, leading to long and unpredictable scan times [6]. Novel techniques have been introduced to correct for respiratory motion that are more time efficient, i.e. self-navigation using k-space [7, 8], 2- and 3-dimensional (2D and 3D) image navigators, and image-based motion models [9–15].

Cardiac motion is frequently addressed using electrocardiogram (ECG)-based gating. The acquisition window is placed in the part of the cardiac cycle with the least motion. This short time window is not long enough to acquire the entire k-space in a single heartbeat; therefore, the acquisition is performed in a segmented fashion over multiple cardiac cycles [16]. Alternatively, in free-running techniques, where data are continuously acquired, the acquisition is performed throughout the entire cardiac cycle. The simultaneously recorded ECG signal enables k‐space trajectory sorting into bins for reconstruction [16, 17]. Another approach is to prospectively estimate cardiac phases from the self-gating signal directly as has been shown in several studies [18].

Similar to MRI, respiratory and cardiac motion are the two main sources of image quality degradation in cardiovascular PET. Again, the ECG signal can be used to reconstruct images based on the PET data that are acquired during the part of the cardiac cycle with the least motion, typically the end-diastolic phase. A disadvantage of cardiac gating is that it results in a considerable signal-to-noise ratio (SNR) loss, since PET counts that are acquired in other parts of the cardiac cycle need to be discarded. Respiratory motion signals can be derived from external sources such as a respiratory belt [19], temperature-based sensing [20], infrared cameras to detect motion of markers placed on patients’ abdomen, [21] data driven gating algorithms [22] or PET data counts since the number of counts can depend considerably on the fraction of the liver within the PET field of view (FOV) in case of PET scan with ^18^F-FDG [23] or potentially ^18^F-FCH [24] as the tracer. A limitation of recording respiratory motion using an external sensor is the indirect estimation of the respiratory motion of the heart from the motion of the thorax. In general, these techniques also have the disadvantage that PET images are reconstructed based on data that are acquired during end expiration, thereby only using approximately 30% of the total counts and thus significantly reducing the SNR. Integrated PET/MRI offers the unique advantage to leverage superior MRI-based motion information for PET motion correction.

Munoz et al. [25] developed a technique that utilizes a 2D image navigator (iNAV) based sequence to perform 3D whole-heart MRI and PET motion correction. Using this technique, three-dimensional motion fields are generated for non-rigid transformation of images from various respiratory phases to a reference position (usually end-expiration). Consequently, all the PET data that are acquired simultaneously with the 3D whole-heart sequence can be used for respiratory motion-corrected PET reconstruction of the heart. However, since the motion information from the navigator is available only for a short duration of the complete PET/MRI scan, i.e., during the 3D whole-heart sequence, only PET data acquired while the navigator is active can be corrected for respiratory motion. This equates to reconstructing PET images based on data acquired during approximately 10 min of the total scan time while the remaining PET data is discarded, which substantially decreases the SNR compared to non-motion corrected reconstructions of the entire PET data set. Therefore, the iNAV-based method to prevent image quality degradation due to respiratory motion cannot be easily combined with cardiac gating, since cardiac gating would lead to additional SNR loss.

In the present study, we describe an extension of the technique developed by Munoz et al. [25]. This extended method allows respiratory motion correction of the heart using the complete acquired PET dataset, utilizing a combination of the 2D iNAV-based coronary MR angiography (CMRA) sequence and a respiratory belt. By using the complete PET dataset, in contrast to the previously published iNAV-based method, we expect that this framework can also be combined with cardiac gating to prevent image degradation due to cardiac motion because the total number of PET counts is much higher.

We will test the proposed framework using cardiovascular PET/MRI data of 19 patients. First, we hypothesize that the proposed framework will lead to an increase in the maximal PET signal intensity in volumes of interest due to less respiratory-motion induced blurring compared to reconstructions without motion-correction. Second, we hypothesize that the SNR in our proposed framework will not be compromised compared to non-motion corrected images. Last, we will explore whether the proposed framework can be combined with cardiac gating.

## Methods

### Patient inclusion

Twelve oncology patients were included in the study that were scheduled for a ^18^Fluoride-fluorodeoxyglucose (^18^F-FDG)-PET/CT for regular clinical care. Each patient was injected with 3 MBq/kg body weight of the tracer. These patients underwent an additional PET/MRI examination. Of the 12 patients injected with ^18^F-FDG, 2 patients did not complete the scan due to discomfort and therefore these patients were excluded. Data from 2 more scans could not be used due to corruption of the listmode file and distortion of the respiratory signal. Furthermore, another 15 patients suffering from coronary artery disease (CAD) that underwent an ^18^F-FCH cardiac PET/MRI examination were also included in the study. Each patient was injected with 4 MBq/kg body weight of the tracer. Of the 15 patients injected with ^18^F-FCH, data from 2 patients could not be used due to an erroneously highly undersampled CMRA and a distorted belt respiratory signal, respectively. The tracer was administered extravascularly for another 2 patients, therefore the data from these patients was also not analyzed.

The approval for both studies was obtained from the Institutional Review Board and the local medical ethical research committee (METC Academisch Ziekenhuis Maastricht) with reference numbers METC 164156 and 162043 respectively. All patients provided written informed consent.

### PET/MRI examination

Each patient was scanned with an integrated 3T PET/MRI scanner (Biograph mMR, Siemens Healthineers, Erlangen, Germany). The patients were scanned in headfirst supine position with the 6-channel body matrix coil and the 12-channel spine radiofrequency coils (Siemens Healthineers, Erlangen, Germany). A Dixon-based attenuation map (µ-map) was acquired at the end-expiration position during a breath-hold for attenuation correction of the PET data [26]. To prevent truncated MR-based attenuation correction maps due to the smaller MRI FOV, B_0_ homogenization using gradient enhancement (HUGE) was used [27]. Subsequently, a 3D-CMRA sequence with a 2D iNAV was acquired, which consists of a 3D spoiled gradient echo sequence with a fully sampled golden-angle step Cartesian trajectory with spiral profile ordering such that one spiral-like interleaf is acquired per heartbeat [25]. Sequence parameters include FOV = 304 × 304 × 96–104 mm^3^ (based on the patient’s anatomy, the field of view can differ slightly and therefore a range is provided in the feet-head direction), acquired resolution = 1.0 × 1.0 × 2.0 mm^3^, flip angle = 15°, repetition time (TR) = 3.72 ms, echo time (TE) = 1.7 ms, patient-specific trigger delay, preferably targeting the end-diastole phase, with an acquisition window ranging between 90 and 130 ms (corresponding to 24–32 lines per spiral interleaf). Just before the 3D CMRA acquisition window, a 2D iNAV is acquired which provides a low-resolution image of the heart in coronal view. As part of the imaging protocol, other cardiac MRI sequences, including cardiac cine MRI are acquired as well. Simultaneously, listmode cardiac PET data is acquired and the respiratory signal is recorded via the respiratory belt for the entire duration of the scan. PET image reconstruction was performed using e7 Tools (Siemens Healthineers, Knoxville, TN, USA), using the ordinary Poisson-ordered subset expectation maximization (OP-OSEM) algorithm with 3 iterations and 21 subsets [28]. Images were reconstructed with a voxel size of 2.08 × 2.08 × 2.03 mm^3^ and a matrix size of 344 × 344 × 127. For PET attenuation correction the MR-based (Dixon) µ-maps were used which provided segmentation of air, lung, fat, and soft tissue.

### CMRA motion correction

Respiratory motion in the left–right (LR) and feet head (FH) direction is estimated using the position of the left ventricle of the heart in the iNAV images for each heartbeat. Based on the FH position, the CMRA data is allocated to certain respiratory phases (bins). In our study, we used 4 bins. K-space data inside each bin is corrected to the center of the bin using the FH and LR position estimates derived from the iNAV. Afterwards, each bin is reconstructed. Using the end-expiration bin as a reference, 3D non-rigid deformation fields are generated based on free form deformations using voxel based normalized mutual information as a similarity measure [29, 30]. These deformation fields are subsequently applied to transform each bin to the end-expiration position generating the motion-compensated CMRA image [25].

### PET motion correction

**Fig. 1:**
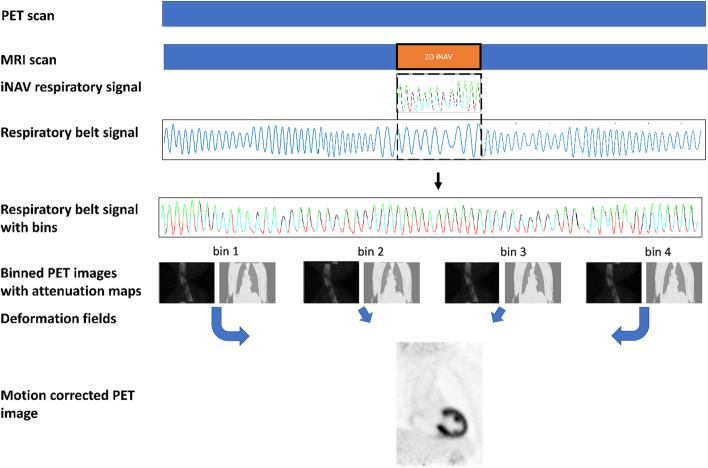
PET and MRI data are acquired simultaneously. The 2D iNAV is only available for part of the complete examination (i.e. during the CMRA acquisition). The feet-head (FH) motion signal is derived from the iNAV to generate the iNAV respiratory signal. The different colors (green, black, red, and cyan) each represent the bin in which the data acquired during this time window will be allocated. The breathing signal from the respiratory belt (thoracic motion) is available for the entire duration of the scan. By co-registration of the iNAV and belt respiratory signal in the period in which they are acquired simultaneously, the respiratory signal in the entire time period can be used to bin the PET data of the entire PET examination. The µ-map is deformed using the inverse motions fields to match the position of each bin. The deformed µ-map is used to reconstruct PET data for each bin. Next, these PET images are transformed to the end-expiration position using the motion fields again and combined to create the final motion-corrected PET image

**Fig. 2:**
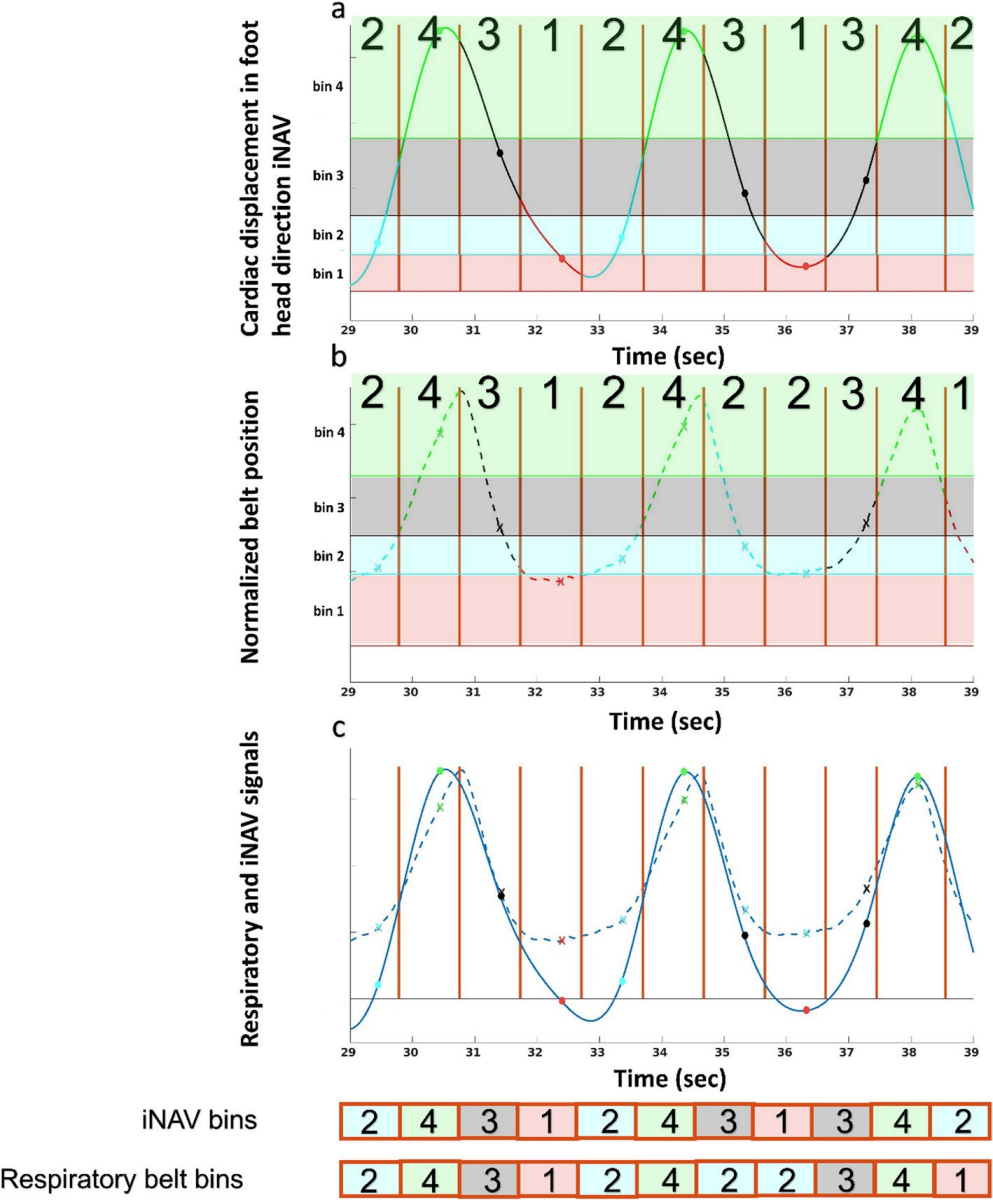
**a** Feet head motion recorded by the iNAV. **b** Thoracic motion recorded with the respiratory belt. **c** Overlapping signal from the iNAV (**a**) and the respiratory belt (**b**). The solid vertical lines represent the R-peak in the ECG signal. The dots on curve in sub-plot a represent the time points at which the iNAV was acquired in the R–R wave. The crosses on curve in sub-plot b represent the corresponding time points signal from the respiratory belt. The shaded horizontal regions in panel a and b represent the 4 bins. All PET data acquired in an R-R interval is allocated to one of the 4 bins. The bin to which the data is allocated is based on the displacement of the heart as determined by the iNAV. For example, in panel a, the data in the first panel is allocated to bin 2 as the iNAV acquired for the R–R interval falls within the bin 2 thresholds. The four colors represent the 4 bins. Red represents bin 1, cyan bin 2, black bin 3 and green bin 4

A schematic representation of PET respiratory motion correction is displayed in Fig. 1. The motion correction pipeline was developed in MATLAB (MATLAB (2018) 9.7.0.1190202 (R2019b); Natick, Massachusetts: The MathWorks Inc.). To utilize the entire PET dataset acquired during the complete PET/MRI exam, the respiratory signal from the respiratory belt is used. The respiratory signal from the iNAV, respiratory belt, and the bins to which the data are allocated are visualized in Fig. 2. The two signals (FH motion signal derived from the iNAV and respiratory belt signal, respectively) are synchronized as described in more detail below. For the time period where iNAV signal is acquired (CMRA acquisition), the respiratory signal from the belt is initially divided into the same bins (bin start and end time) as those that were used for the CMRA motion correction. The scale on the y-axis of the respiratory belt signal is adjusted to the same order of magnitude as the iNAV signal, which allows the use of the same bin thresholds (FH displacement ranges that determine to which bin the acquired data are allocated) for CMRA motion correction and the respiratory belt signal binning as a first input in an iterative procedure. The iNAV signal is acquired every heartbeat. Therefore, using ECG R-R wave intervals as reference, corresponding bins from the iNAV and respiratory belt are compared to determine the bin match percentage between the 2 signals. Subsequently, the bin threshold ranges for the respiratory belt signal are manually adjusted to provide the highest bin match percentage between the 2 signals. Once the final threshold ranges are determined, they are used to bin the respiratory belt signal for the remaining part of the PET acquisition.

Based on the binning threshold ranges, PET counts are divided into four bins. For each bin, a listmode file is created. PET images, µ-maps and MRI-derived deformation fields have different dimensions. To enable application of the deformation fields to µ-maps and PET images the data were interpolated and zero-padded to the largest dimension in each direction, resulting in matching matrices where each point represents the same physical position. The µ-map is deformed to reconstruct 4 µ-maps that match the position of each bin using the inverse of the deformation fields that were generated previously to reconstruct the CMRA. To reconstruct the cardiac gated PET images, data for each bin is cardiac gated to include only the PET data that was acquired simultaneously with the CMRA acquisition window (acquired preferably in end-diastolic phase). Using the listmode files and the µ-maps, PET data is reconstructed for each bin as described above. Finally, using the same deformation fields, each PET image is transformed to the reference position (end-expiration) and, subsequently, combined (corresponding pixels from each of the four reconstructed bins are averaged) to create the final motion-corrected PET image.

### Image analysis

For each patient in the ^18^F-FDG and ^18^F-FCH study, PET data were reconstructed in four ways: a dataset with neither respiratory nor cardiac motion correction (NMC), a dataset with only respiratory motion correction using the framework provided in this manuscript (MC), a dataset with no respiratory motion correction but with end-diastolic gating (NMC_G) and a dataset with respiratory motion correction using the framework provided in this manuscript combined with end-diastolic gating (MC_G).

**Fig. 3:**
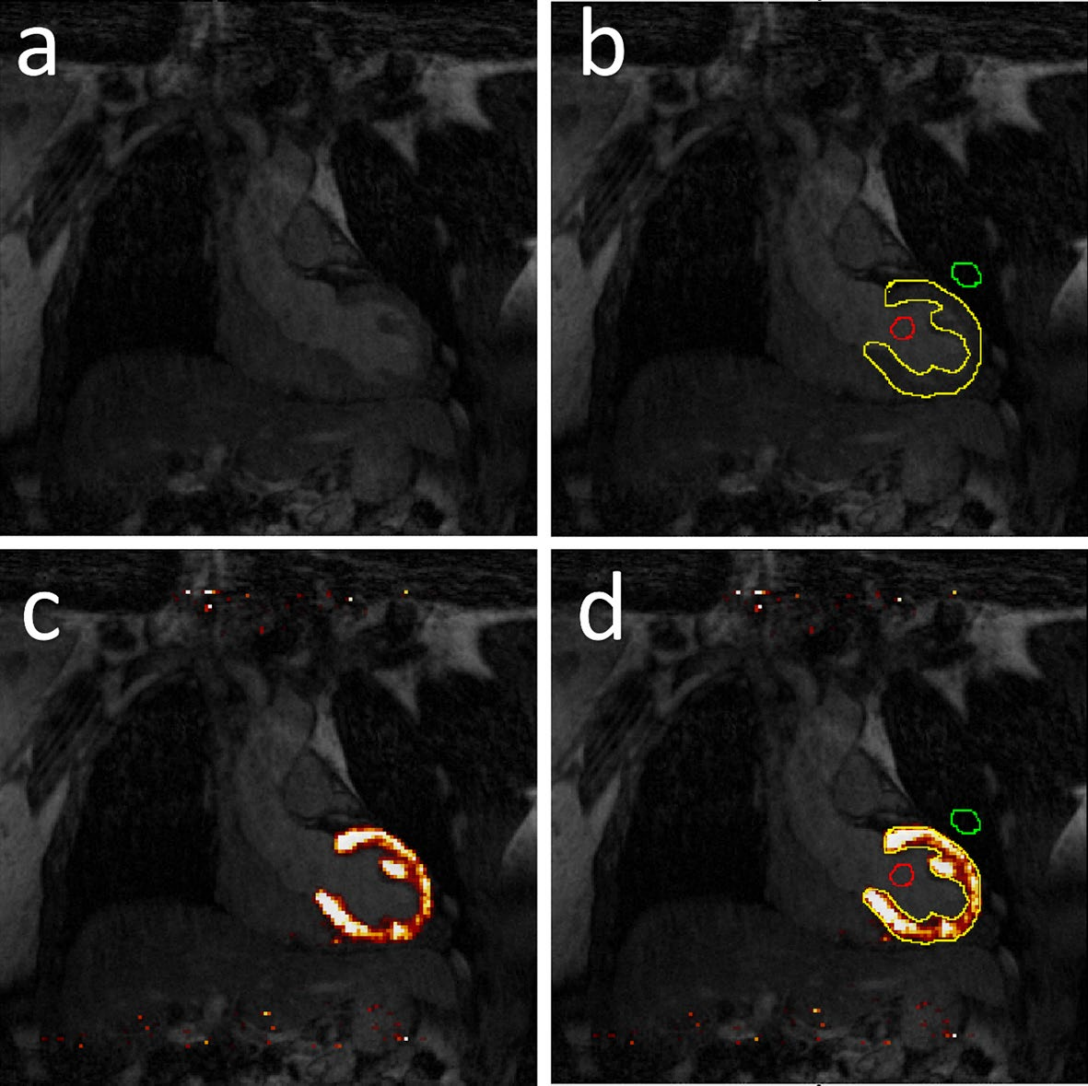
MR and ^18^F-FDG PET/MR images of the myocardium. **a** CMRA image, **b** CMRA image with the volumes of Interest (VOIs) that encompasses the left ventricular wall (yellow), a VOI in the middle of the left ventricular cavity (red) and a VOI in the lung close to the myocardium (green) **c** shows a color overlay of the PET image projected on the CMRA image. **d** PET/MR image with the three VOIs

For the ^18^F-FDG datasets, volumes of interest (VOIs) were drawn in the 5 middle CMRA slices delineating the left ventricular wall and the middle of the left ventricle cavity (Fig. 3). The corresponding PET images were overlaid on the CMRA images in color. The VOI on the myocardial wall was used to calculate the maximal myocardial ^18^F-FDG uptake, expressed as maximal Standardized Uptake Value (SUV_max_). The SUV_max_ is defined as the maximum voxel activity within the VOI. The myocardial SUV_max_ were normalized to the mean blood pool activity concentration obtained from the VOI in the center of the left ventricular cavity (SUV_mean_ blood pool). The resulting normalized values were expressed as maximum target-to-background ratio (TBR_max_). Blurring of the image due to respiratory motion is expected to reduce the myocardial SUV_max_ and TBR_max_ values. Therefore, higher TBR_max_ values are expected for the respiratory motion-corrected images. We also calculated the SNR, defined as the ratio of the signal intensity in the VOI in the myocardial wall and the standard deviation of the SUV in a VOI with low uptake (lung) as the inhomogeneous uptake in the myocardium does not allow to quantify the noise reliably in the VOI in the myocardium [31]. The SNR is calculated using the following equation.

$$SNR = \left( {Mean\;of\;the\;myocardium\;VOI} \right)/\left( {Standard\;deviation\;of\;the\;lung\;VOI} \right)$$

The statistical noise in PET images is related to the number of PET counts [32]. The motion-corrected images have the same number of PET counts as the non-motion corrected images and therefore these images are expected to have a similar SNR. Cardiac gating leads to a large reduction in the number of PET counts which reduces the SNR. Therefore, we expected a higher SNR for the MC compared to the MC_G images and also a higher SNR for the NMC versus NMC_G image.

To qualitatively assess the PET images, line profiles were drawn through a high tracer uptake region (myocardial walls) for the ^18^F-FDG dataset and these line profiles were visually inspected.

For the ^18^F-FCH datasets, a volume of interest was drawn that delineates a vulnerable atherosclerotic lesion, which was selected based on a vascular section with a vulnerable plaque, defined as a plaque with a fibrous cap thickness of < 70 µm on optical coherence tomography (OCT). The OCT slice position of the vulnerable plaque was identified on the CMRA images, using vessel side branches as landmarks, in consensus by a cardiologist (BR) and nuclear medicine physician (JP). Another VOI was drawn in the left ventricle cavity. The CMRA images were fused with the corresponding PET images. The TBR_max_ was determined as described above. Since the small size of the atherosclerotic lesions and low ^18^F-FCH uptake in the plaques prevented accurate SNR assessment, a VOI in the liver was drawn to calculate the SNR values for the ^18^F-FCH datasets. To calculate the SNR, the mean value of the signal from the liver VOI was used while the standard deviation of the VOI in a low uptake region (lung) was used to determine the noise since inhomogeneous uptake in the liver prevented accurate noise assessment (Additional file 1: Figure Appendix A). The VOI in the liver was also used to assess the SUV_max_ for the ^18^F-FCH datasets. To qualitatively assess the ^18^F-FCH PET images, line profiles were drawn through a high tracer uptake region (at the lung-liver interface) and these line profiles were visually inspected.

### Statistical analysis

The SUV_max_, TBR_max_ and SNR values for the 4 reconstructions were compared using a repeated measures ANOVA test with post hoc tests. The values are presented as the mean ± standard error (SE) of the measurements. A *p*-value < 0.05 was considered statistically significant.

## Results

Eight patients were injected with a mean dose of 232.6 ± 51.2 MBq of ^18^F-FDG as part of their regular clinical care. The mean duration between the injection and the start of the PET/MRI research scan was 111 ± 21 min and the PET scan lasted 26.0 ± 5.0 min on average.

Eleven patients were injected with a mean dose of 282.3 ± 33.5 MBq of ^18^F-FCH. On average the patient was injected with the tracer 2.0 ± 0.5 min after the start of the scan. In one patient, the scan started 11 min and 12 s after the tracer injection. The average scan duration was 75 ± 10.7 min. The duration of the CMRA sequence with the iNav was approximately 10 min in both ^18^F-FDG and ^18^F-FCH datasets. For the ^18^F-FDG study, the bin match percentage (mean ± SD) between the iNav and the respiratory belt where they overlap was 70.8 ± 6.8% while for the ^18^F-FCH datasets the average was slightly higher at 75.8 ± 2.6%.

**Fig. 4:**
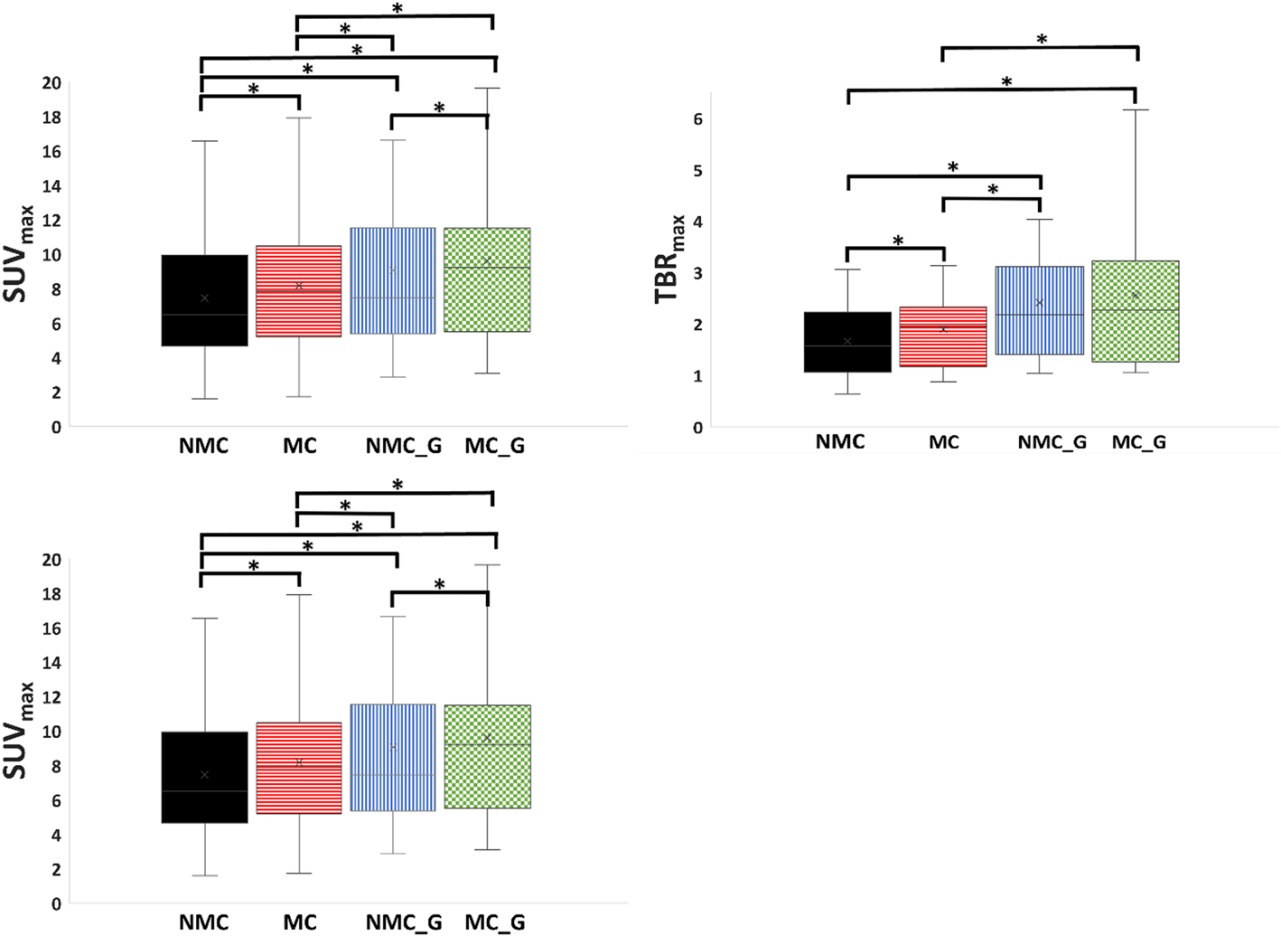
Box plots of the SUV_max_ **a** TBR_max_ **b** and SNR **c** for each of the four reconstructions (NMC, MC, NMC_G and MC_G) for the 19 datasets included in this study. The ‘x’ inside the box represents the mean value, the horizontal line the median value, the box represents the 1st and 3rd quartile. The ‘whiskers’ show the lowest and highest values and the asterisk (*) shows significant difference between two comparisons

The mean SUV_max_, TBR_max_ and SNR values are presented in Fig. 4 and Table 1.

**Table 1:** Table showing the mean and standard error values of each of the four reconstructions for each of the three quantitative parameters assessed in this study

SUV_max_	NMC	MC	NMC_G	MC_G
Mean ± SE	7.5 ± 1.0	8.2 ± 1.0	9.1 ± 1.2	9.6 ± 1.3
*p*-value in comparison with NMC	–	< 0.01	< 0.01	< 0.01
*p*-value in comparison with MC	< 0.01	–	0.02	< 0.01
*p*-value in comparison with NMC_G	< 0.01	0.02	–	0.02
*p*-value in comparison with MC_G	< 0.01	< 0.01	0.02	–
*TBR*_*max*_				
Mean ± SE	1.2 ± 0.2	1.9 ± 0.2	2.4 ± 0.3	2.6 ± 0.3
*p*-value in comparison with NMC	–	< 0.01	< 0.01	< 0.01
*p*-value in comparison with MC	< 0.01	–	0.03	< 0.01
*p*-value in comparison with NMC_G	< 0.01	0.03	–	0.16
*p*-value in comparison with MC_G	< 0.01	< 0.01	0.16	–
*SNR*				
Mean ± SE	72.4 ± 10.5	68.8 ± 10.5	47.1 ± 6.3	45.3 ± 7.2
*p*-value in comparison with NMC	–	0.3	< 0.01	< 0.01
*p*-value in comparison with MC	0.3	–	< 0.01	< 0.01
*p*-value in comparison with NMC_G	< 0.01	< 0.01	–	0.6
*p*-value in comparison with MC_G	< 0.01	< 0.01	0.6	–

The *p*-values from the comparison between different reconstructions are also included in the table

The pair-wise comparison showed that the motion corrected (MC) datasets have significantly higher SUV_max_ and TBR_max_ values compared to the non-motion corrected (NMC) datasets (8.2 ± 1.0 vs 7.5 ± 1.0, *p* < 0.01 and 1.9 ± 0.2 vs 1.2 ± 0.2, *p* < 0.01, respectively). In addition, the SUV_max_ and TBR_max_ of the motion corrected and gated (MC_G) reconstructions were also higher than that of the non-motion corrected but gated (NMC_G) datasets, although for the TBR_max_ this difference was not statistically significant (9.6 ± 1.3 vs 9.1 ± 1.2, *p* = 0.02 and 2.6 ± 0.3 vs 2.4 ± 0.3, *p* = 0.16 respectively). The highest SUV_max_ and TBR_max_ values were observed for the motion-corrected and gated reconstruction. The respiratory motion-correction did not lead to a change in the signal to noise ratio since there was no statistically significant difference in the SNRs of the MC versus NMC and MC_G vs NMC_G dataset. As expected based on the large reduction in PET counts due to cardiac gating, the reconstructions with cardiac gating had a significantly lower SNR.

**Fig. 5:**
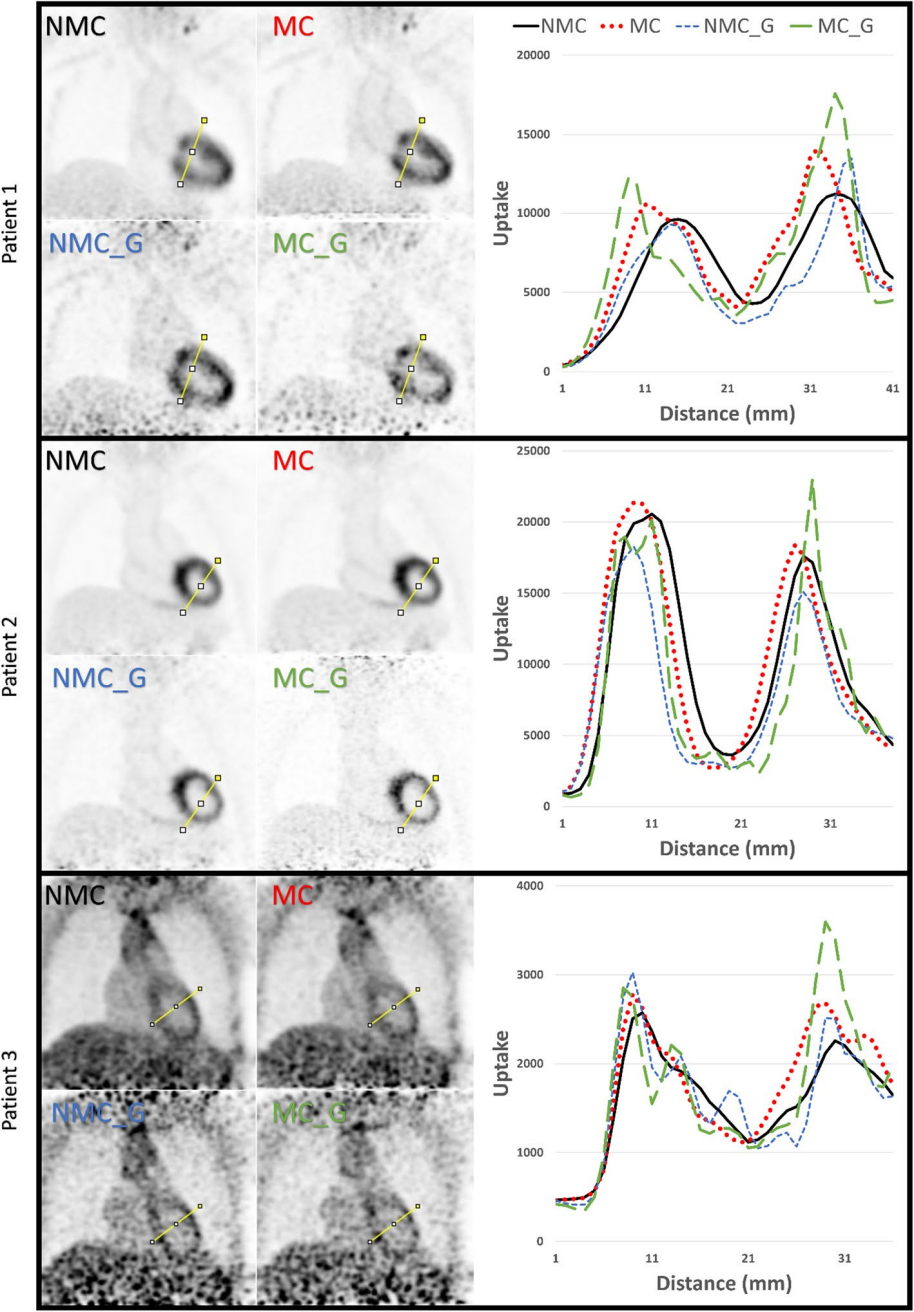
Line profiles (column on the right) through the myocardium of the four PET reconstructions (NMC, MC, NMC_G and MC_G) of three patients injected with ^18^F-FDG are shown. The data for the line profiles were acquired at the same location for each reconstruction per patient (yellow lines). The line profiles represent the ^18^F-FDG uptake values for each reconstruction (black = no motion correction, red = motion corrected no gating, blue = no motion correction but gating and green = motion corrected and gated)

For 3 of the 8 patients injected with ^18^F-FDG, the four different reconstructions (NMC, MC, NMC_G and MC_G) are shown in Fig. 5. The reconstructions of the remaining patients are presented in Additional file 1: Figure Appendix B. The ^18^F-FDG uptake was recorded for each reconstruction at the same location (yellow line) and was plotted against distance (line profiles). The line profiles for each reconstruction show 2 distinct peaks, each representing a myocardium wall (high uptake region) and the region in between representing the blood pool (low uptake region). In most cases, the peak height is higher for MC as compared to NMC while maintaining similar values for minimum blood pool uptake. The introduction of gating reduces the peak width for the NMC and MC datasets. The NMC and MC images are less noisy than the NMC_G and MC_G images and the line profiles of the non-gated images are therefore also smoother.

**Fig. 6:**
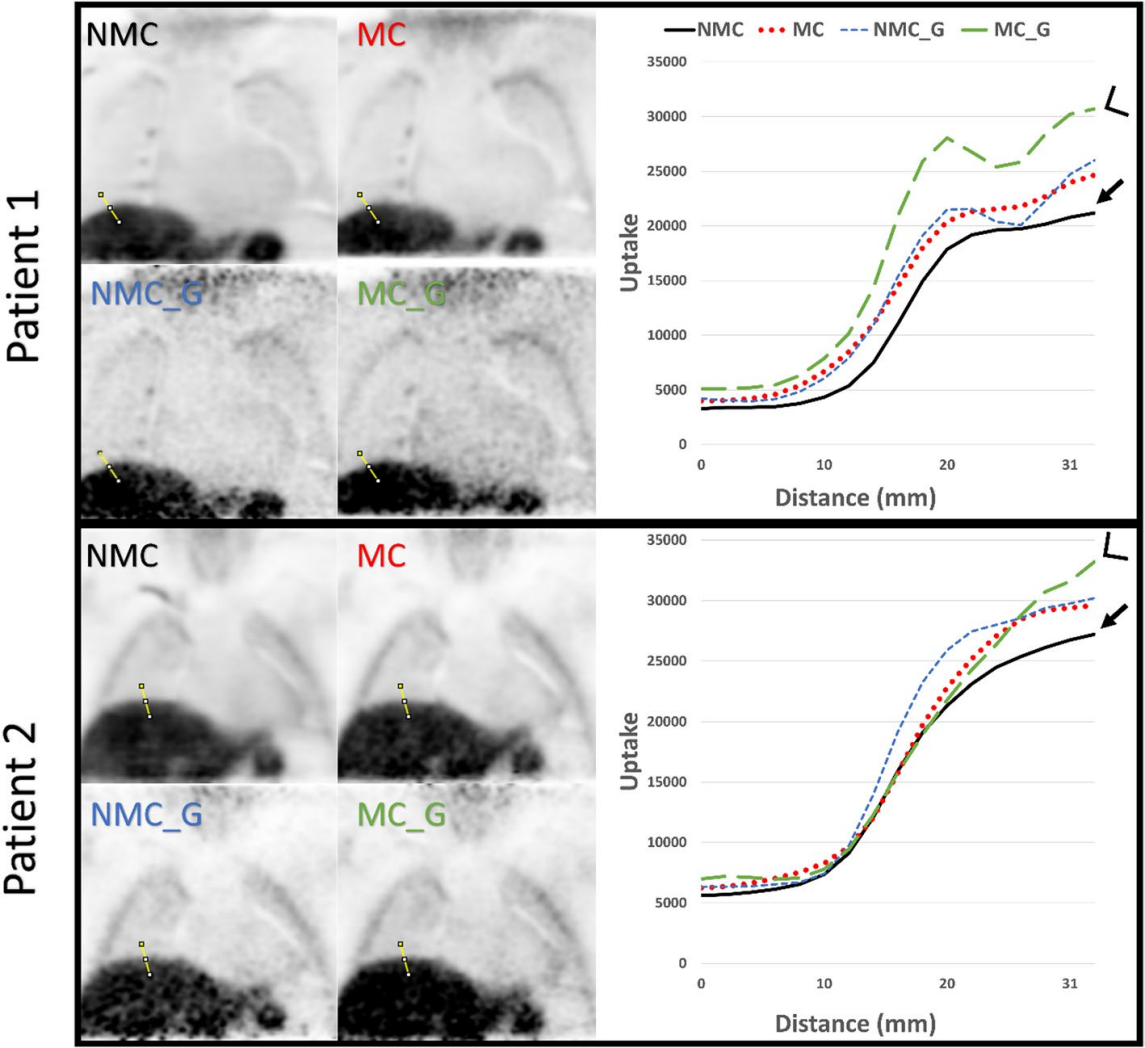
Line profiles (column on the right) on the liver-lung interface of the four PET reconstructions (NMC, MC, NMC_G and MC_G) of two patients injected with ^18^F-FCH are shown. The data for the line profiles were acquired at the same location for each reconstruction per patient (yellow lines). The line profiles represent the ^18^F-FCH uptake values for each reconstruction (black = no motion correction, red = motion corrected no gating, blue = no motion correction but gating and green = motion corrected and gated). The arrow in the plots indicates the maximum uptake value for the no motion correction reconstruction in the liver and the arrow heads indicates the higher uptake value of the reconstruction with motion correction and cardiac gating

For 2 of the 11 patients injected with ^18^F-FCH, the four different reconstructions (NMC, MC, NMC_G and MC_G) are shown in Fig. 6. The reconstructions of the remaining patients are presented in Additional file 1: Figure Appendix C. The line profiles show that the NMC images typically show the lowest uptake in the liver (arrows), while the highest uptake is observed for the combination of respiratory motion-correction and cardiac gating (arrow heads). Again, the cardiac gated image reconstructions are more noisy.

## Discussion

We presented a pipeline for extended MRI-based respiratory motion correction of cardiac PET images for hybrid PET/MRI. The new pipeline has the advantage that none of the cardiac PET data from the entire PET examination is discarded for respiratory motion correction. Indeed, we demonstrated that the motion-corrected PET images of cardiovascular patients by the framework that we proposed were not compromised in SNR compared to the images that were reconstructed without motion correction. The gated as well as the non-gated motion corrected PET images showed higher SUV_max_ values than their non-motion corrected counterparts, which indicates that the motion correction pipeline reduces blurring of the images due to respiratory motion. We also showed that the respiratory motion correction can be combined with cardiac gating. The images that were reconstructed using a combination of respiratory motion correction and cardiac gating had the highest SUV_max_ values.

The most common method to prevent blurring of cardiac PET images due to respiratory motion is respiratory gating. The disadvantage of this method is that a large amount of the PET counts are discarded which reduces the SNR, while in the motion correction framework that we have presented here, the SNR was not affected.

As expected, the cardiac gated reconstructions were noisier and, therefore, had a significantly lower SNR, since these images are reconstructed using only those PET counts that are acquired in the part of the cardiac cycle with the least motion. The SUV_max_ values of the reconstruction using respiratory motion correction and cardiac gating were the highest, since these images were the least affected by blurring due to respiratory and cardiac motion although we cannot exclude that more noise in these images can also lead to an increased SUV_max_.

TBR_max_ is an important parameter in quantitative PET, since it represents the maximal uptake value normalized to blood activity concentration. Our study showed that the TBR_max_ value was higher for the motion-corrected images, although for the gated images this did not reach statistical significance. Again, the highest TBR_max_ values were observed for the framework that combined respiratory motion correction and cardiac gating. Future studies should investigate whether the higher SUV_max_ and TBR_max_ would improve the identification of small lesions or pave the way for reduced radiation dose or scan times.

The qualitative analysis of the line profiles was in line with the quantitative findings, since for most patients higher peak values were observed in the line profiles for the MC versus the NMC datasets. The introduction of cardiac gating reduced the width of the peaks in the ^18^F FDG datasets and increased the signal intensity in the liver in the ^18^F-FCH datasets.

In our study, 2 oncology patients did not complete the PET/MRI scan due to patient discomfort. Note that these oncologic patients underwent an additional PET/MRI scan for research purposes after a clinical PET/CT scan, which added to the discomfort of the patients. The presented method is an extension of the pipeline that was previously presented by Munoz et al. [14]. Munoz et al. used only the iNAV-based portion of the protocol for PET motion correction. They showed increased sharpness as compared to non-motion corrected data and reduced noise as compared to the gated dataset. Since this previous method can use only the PET data that are acquired while the iNAV is running (≈ 10 min), the number of PET counts is low compared to the total PET counts for a complete scan. Therefore, the number of PET counts for this method is typically too low to combine respiratory motion correction with cardiac gating, since cardiac gating further reduces the total number of counts. The framework introduced in the present study extends the use of iNAV for motion correction using the respiratory belt allowing respiratory motion correction without any count loss, resulting in less noisy images, and enabling the combined application of respiratory motion correction and cardiac gating for PET image reconstruction. Our proposed framework is not necessarily dependent on the respiratory belt. Any technique that allows the acquisition of the breathing signal such as the data-driven approach [33] can potentially be utilized, which would remove the dependence on external hardware.

In a recent development, Munoz et al. [34] added anatomically guided PET reconstruction using the simultaneously acquired CMRA images to their pipeline. The CMRA images are used to identify voxels from the same tissue (thus voxels with a similar tracer uptake on PET images) to apply a smoothing filter on these voxels in the PET image. This resulted in a 143% increase in contrast between the blood pool and myocardium and a 16% decrease in image noise. The current pipeline could also be extended with anatomically guided PET which may lead to further improvement of SNR.

Robson et al. [23] used the change in PET data counts due to the fraction of liver in the field of view to generate the breathing signal and perform respiratory gating, and the ECG signal for cardiac gating, before spatially transforming them to a single motion-corrected PET reference frame. Similarly, Mayer et al. [35] used a respiratory belt and ECG signal to create a cardiorespiratory motion model and used it to create a motion-corrected PET image. A common attribute of both these techniques is simultaneous PET/MRI acquisition. There is usually a ground truth signal such as the MR self-navigation and signals from external sensors such as the respiratory belt, ECG, or motion-detecting cameras. Some techniques simply rely on the information provided by the external sensors only (respiratory belt and ECG) to perform cardiorespiratory gating. The advantage of the framework introduced in the present study is that it utilizes a 2D image navigator. As the navigator is placed on the heart, it records the actual motion of the heart and not an indirect measure such as the respiratory belt measuring the expansion and relaxation of the thoracic cavity. Also, techniques that use PET counts to extract the respiratory signal will not work for tracers like ^18^F-NaF which are not taken up in the liver. Küstner et al. [36] also implemented a technique to correct the complete PET data for motion. In a simultaneous PET and MRI acquisition, they used a 90-s-long MR sequence with self-navigation together with external sensors to measure respiratory motion, to create a surrogate signal. This surrogate signal was trained based on the 90-s MR self-navigation signal to predict the breathing motion for the remaining duration of the scan. Dual gating (respiratory and cardiac) was performed using the surrogate signal in combination with the ECG signal to correct for respiratory and cardiac motion. They reported a 22% improvement in lesion quantification, 64% in lesion delineation and 23% in the diagnostic confidence level. The training method implemented by Küstner et al. [36] could be introduced in the current pipeline for further improvement. Specifically, the time window during which both the iNAV and respiratory belt data are acquired can be utilized as a training period to estimate a surrogate signal for the remaining duration of the PET scan. By incorporating this technique, it may be possible to further improve the respiratory motion correction and thus enhance the overall quality of the reconstructed PET images.

In the current implementation of our framework, we use only one cardiac gate, thereby neglecting PET data that were acquired outside the end-diastole phase for the cardiac gated images. The next step in the development of this framework could be multiple gates such that none of the acquired PET data is discarded. The iNAV has a low temporal resolution since it is acquired once per heartbeat, and therefore all PET data acquired within one R-R duration is assigned to a single bin, although the position of the heart in the foot-head direction may change within one heartbeat. The respiratory belt has a higher temporal resolution and therefore, in future studies, it could be investigated whether PET data allocation to bins can be further improved by allocating multiple bins per heartbeat.

The present study has a number of limitations. The PET/MRI scan of the patients injected with ^18^F-FDG was performed approximately 112 min post-tracer injection, surpassing the recommended optimal interval of 45–60 min for imaging. However, since the same data set was used for all 4 reconstructions, this should not affect the comparison between the four reconstructions.

Another limitation of our study was that we found a low uptake of ^18^F-FCH in the coronary plaques, with only 2/11 of the NMC datasets showing a TBR_max_ value of > 1.5, which increased to 5/11 patients after respiratory motion correction. For this reason and because of the small size of coronary plaques, we have calculated the SNR in a volume of interest in the liver for the ^18^F-FCH data, since large uptake in the liver was observed. Additionally, we did not optimize the number of bins in our proposed framework. Further research is warranted to determine the impact of varying the quantity of bins to identify an optimal bin number based on the trade-off between image quality and acceptable-post-processing times. Furthermore, in our otherwise automated pipeline, the thresholds used to bin the PET data were still manually adjusted. This step could be improved by introducing an automated method such as a gradient descent algorithm or principal component analysis. Such a technique is expected to make the threshold adjustment procedure more consistent and reproducible. Moreover, in our pipeline we did not check for changes in breathing pattern during the scan, which could lead to incorrect binning of the PET data. To counter this, a dynamic binning process could be introduced that checks the minimum and maximum amplitude for each breath and bins the PET data accordingly. Alternatively, data acquired during a period with a change in breathing pattern could be discarded.

## Conclusion

In this study, we introduced a novel framework to correct for respiratory motion of cardiac PET/MRI images utilizing a 2D MRI-based image navigator framework and a respiratory belt to ensure the entire PET dataset is used to create the motion-corrected images. Since no data is discarded for respiratory motion correction, we showed that the SNR was not reduced by motion correction. We demonstrated that the proposed framework can be combined with cardiac gating. Our results showed that the extended motion correction method resulted in higher SUV_max_ values compared to reconstructions without motion correction and that the SUV_max_ value could be further improved by combining the proposed method with cardiac gating. Future cardiac PET/MRI studies could include techniques such as the one developed in this study to improve image quality.

### Supplementary Information

**Additional file 1: Appendix A.** MR and ^18^F-FCH PET_MR images of the heart and the liver. **a** CMRA image, **b** CMRA image with the volume of Interest (VOI) in the liver at the liver-lung interface (yellow) and the VOI in the lung to assess the noise (red). **c** Shows a color overlay of the PET image projected on the CMRA image. **d** PET/MR image with the VOIs. **Appendix B.** Line profiles (column on the right) through the myocardium of the four PET reconstructions (NMC, MC, NMC_G and MC_G) of patients injected with ^18^F-FDG are shown. The data for the line profiles were acquired at the same location for each reconstruction per patient (yellow lines). The line profiles represent the ^18^F-FDG uptake values for each reconstruction (black = no motion correction, red = motion corrected no gating, blue = no motion correction but gating and green = motion corrected and gated). **Appendix C.** Line profiles (column on the right) on the liver-lung interface of the four PET reconstructions (NMC, MC, NMC_G and MC_G) of patients injected with ^18^F-FCH are shown. The data for the line profiles were acquired at the same location for each reconstruction per patient (yellow lines). The line profiles represent the ^18^F-FCH uptake values for each reconstruction (black = no motion correction, red = motion corrected no gating, blue = no motion correction but gating and green = motion corrected and gated). The arrow in the plots indicates the maximum uptake value for the no motion correction reconstruction in the liver and the arrow heads indicates the higher uptake value of the reconstruction with motion correction and cardiac gating.

## Data Availability

For ethical reasons, the raw data that we collected cannot be made publicly available. The study was approved by the Medical Ethics Committee of the Maastricht University Medical Center, Maastricht, The Netherlands under the condition that access to the data is granted only to (1) members of the research team, (2) the Medical Ethics Committee members that approved this study, and (3) authorized personnel of the Health Care Inspectorate. Hence, participants did not consent to publicly archive their data. However, requests for anonymous data can be sent to prof. dr. ME Kooi at eline.kooi@mumc.nl.
