# Supplementary material for: Extended MRI-based PET motion correction for cardiac PET/MRI
Source: EJNMMI Phys. 2024 Apr 6;11:36. doi: 10.1186/s40658-024-00637-z (PMC10998820; doi:10.1186/s40658-024-00637-z)
Supplement: Supplementary file 1 — Additional file 1: Appendix A. MR and 18F-FCH PET_MR images of the heart and the liver. a CMRA image, b CMRA image with the volume of Interest (VOI) in the liver at the liver-lung interface (yellow) and the VOI in the lung to assess the noise (red). c Shows a color overlay of the PET image projected on the CMRA image. d PET/MR image with the VOIs. Appendix B. Line profiles (column on the right) through the myocardium of the four PET reconstructions (NMC, MC, NMC_G and MC_G) of patients injected with 18F-FDG are shown. The data for the line profiles were acquired at the same location for each reconstruction per patient (yellow lines). The line profiles represent the 18F-FDG uptake values for each reconstruction (black = no motion correction, red = motion corrected no gating, blue = no motion correction but gating and green = motion corrected and gated). Appendix C. Line profiles (column on the right) on the liver-lung interface of the four PET reconstructions (NMC, MC, NMC_G and MC_G) of patients injected with 18F-FCH are shown. The data for the line profiles were acquired at the same location for each reconstruction per patient (yellow lines). The line profiles represent the 18F-FCH uptake values for each reconstruction (black = no motion correction, red = motion corrected no gating, blue = no motion correction but gating and green = motion corrected and gated). The arrow in the plots indicates the maximum uptake value for the no motion correction reconstruction in the liver and the arrow heads indicates the higher uptake value of the reconstruction with motion correction and cardiac gating. [file 40658_2024_637_MOESM1_ESM.docx]

# Appendix


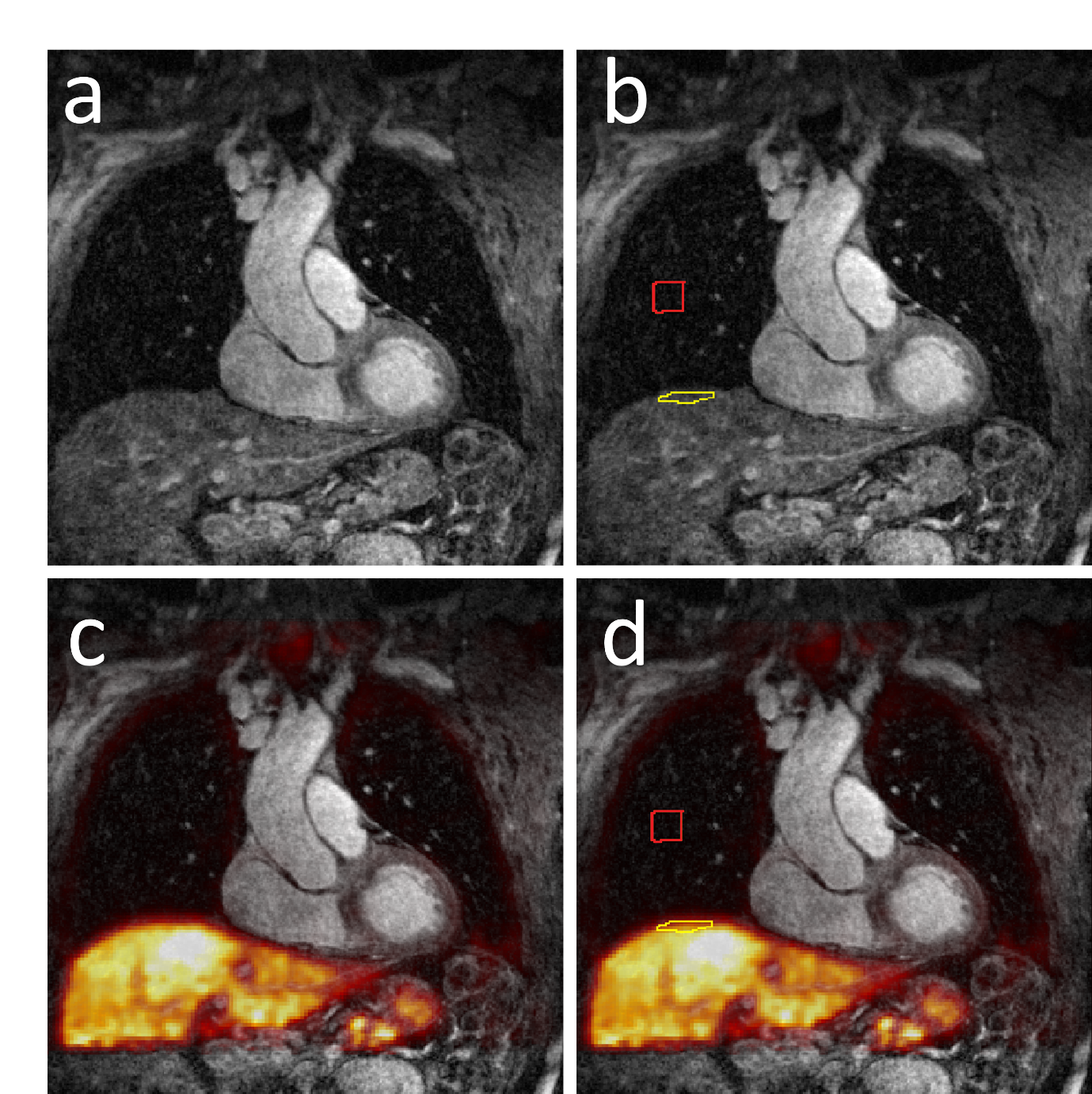


*Appendix A. MR and ^18^F-FCH PET_MR images of the heart and the liver. (a) CMRA image, (b) CMRA image with the volume of Interest (VOI) in the liver at the liver-lung interface (yellow) and the VOI in the lung to assess the noise (red). (c) shows a color overlay of the PET image projected on the CMRA image. (d) PET/MR image with the VOIs.*

*
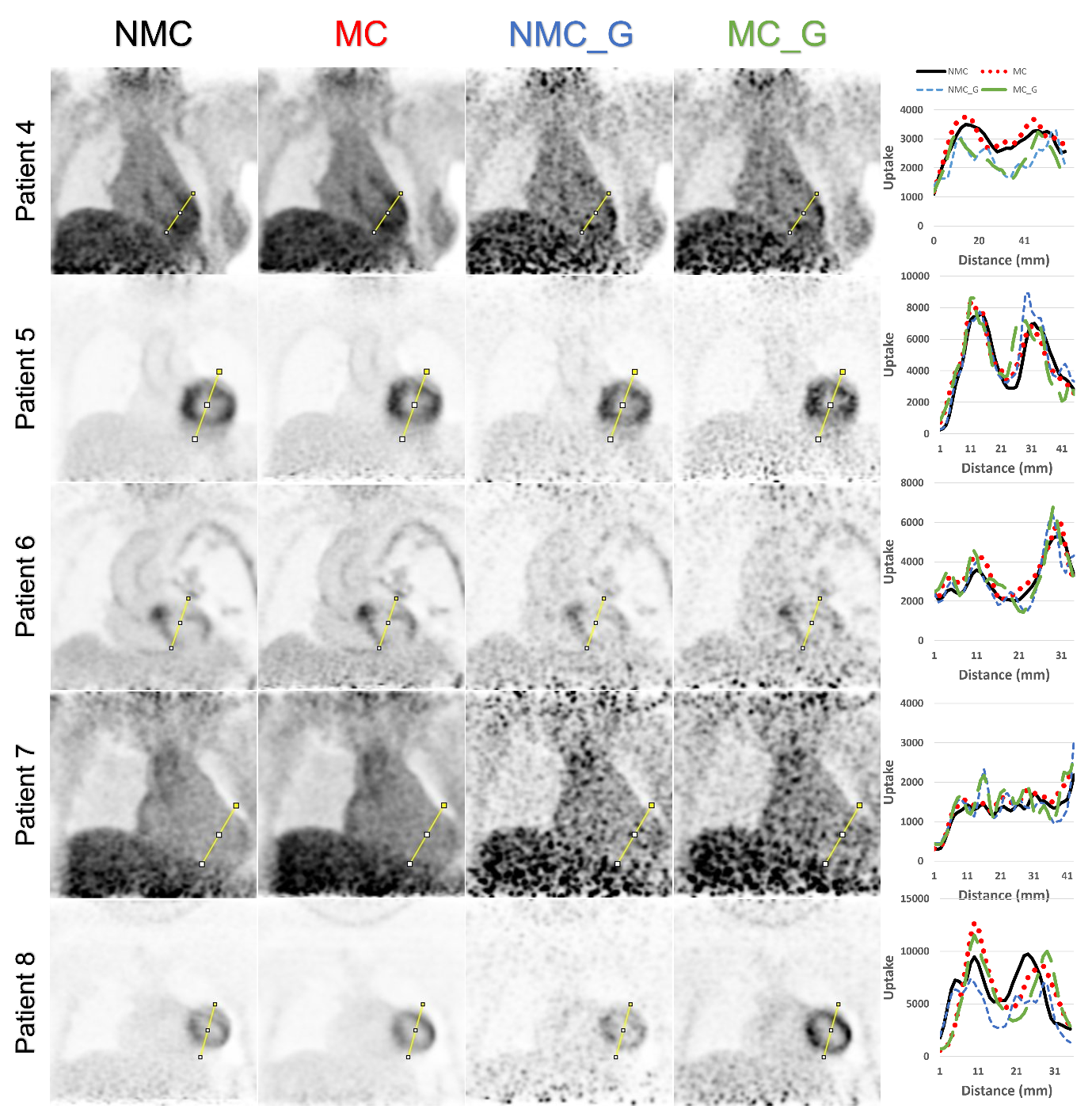
*

*Appendix B. Line profiles (column on the right) through the myocardium of the four PET reconstructions (NMC, MC, NMC_G and MC_G) of patients injected with ^18^F-FDG are shown. The data for the line profiles were acquired at the same location for each reconstruction per patient (yellow lines). The line profiles represent the ^18^F-FDG uptake values for each reconstruction (black = no motion correction, red = motion corrected no gating, blue = no motion correction but gating and green = motion corrected and gated).*
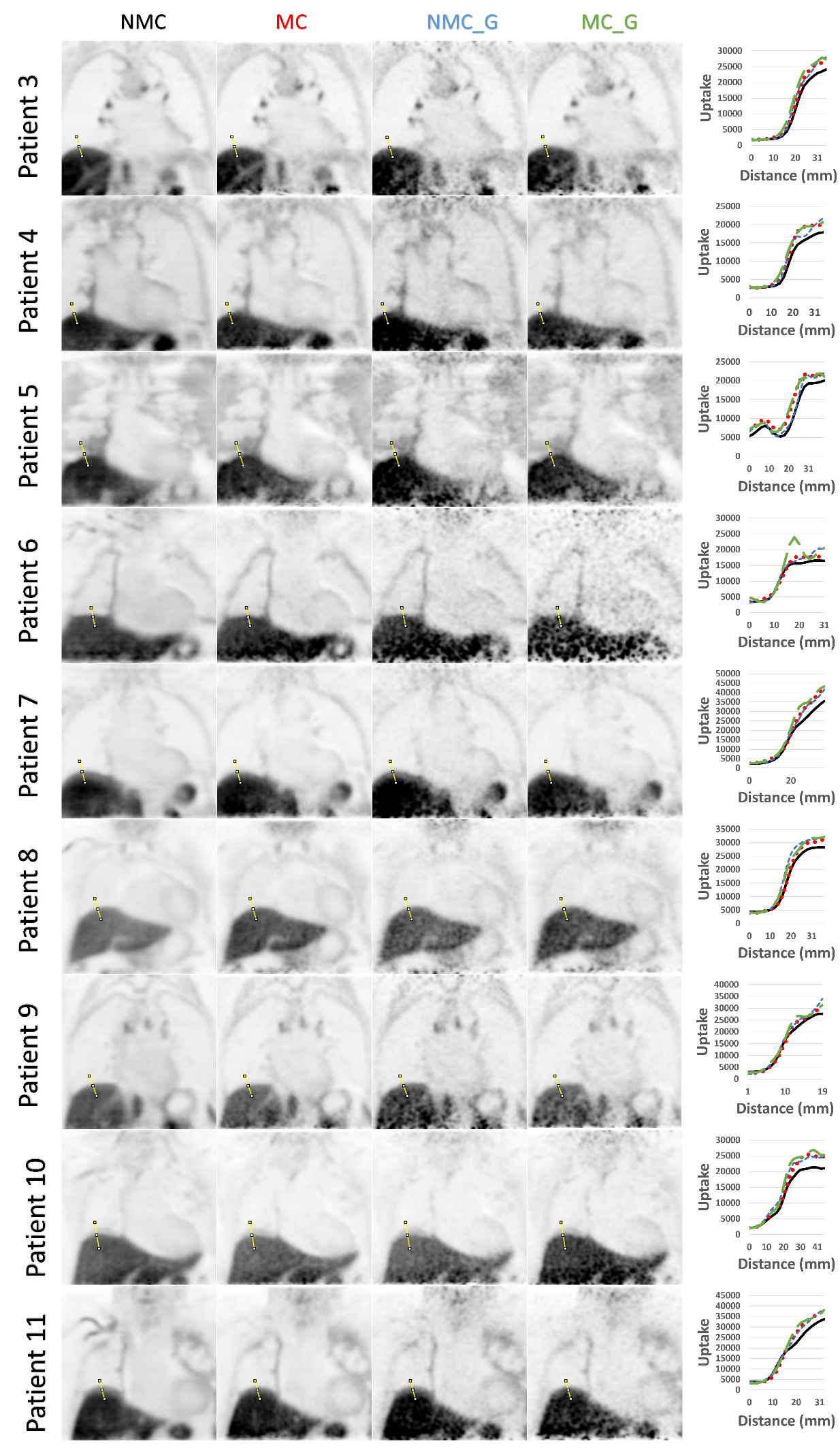


*Appendix C. Line profiles (column on the right) on the liver-lung interface of the four PET reconstructions (NMC, MC, NMC_G and MC_G) of patients injected with ^18^F-FCH are shown. The data for the line profiles were acquired at the same location for each reconstruction per patient (yellow lines). The line profiles represent the ^18^F-FCH uptake values for each reconstruction (black = no motion correction, red = motion corrected no gating, blue = no motion correction but gating and green = motion corrected and gated). The arrow in the plots indicates the maximum uptake value for the no motion correction reconstruction in the liver and the arrow heads indicates the higher uptake value of the reconstruction with motion correction and cardiac gating.*
